# Macrophages and cytokines in the early defence against herpes simplex virus

**DOI:** 10.1186/1743-422X-2-59

**Published:** 2005-08-03

**Authors:** Svend Ellermann-Eriksen

**Affiliations:** 1 Department of Clinical Microbiology, Aarhus University Hospital, Skejby Sygehus, Brendstrupgaardsvej 100, DK-8200 Aarhus N., Denmark

## Abstract

Herpes simplex virus (HSV) type 1 and 2 are old viruses, with a history of evolution shared with humans. Thus, it is generally well-adapted viruses, infecting many of us without doing much harm, and with the capacity to hide in our neurons for life. In rare situations, however, the primary infection becomes generalized or involves the brain.

Normally, the primary HSV infection is asymptomatic, and a crucial element in the early restriction of virus replication and thus avoidance of symptoms from the infection is the concerted action of different arms of the innate immune response. An early and light struggle inhibiting some HSV replication will spare the host from the real war against huge amounts of virus later in infection. As far as such a war will jeopardize the life of the host, it will be in both interests, including the virus, to settle the conflict amicably. Some important weapons of the unspecific defence and the early strikes and beginning battle during the first days of a HSV infection are discussed in this review.

Generally, macrophages are orchestrating a multitude of anti-herpetic actions during the first hours of the attack. In a first wave of responses, cytokines, primarily type I interferons (IFN) and tumour necrosis factor are produced and exert a direct antiviral effect and activate the macrophages themselves. In the next wave, interleukin (IL)-12 together with the above and other cytokines induce production of IFN-γ in mainly NK cells. Many positive feed-back mechanisms and synergistic interactions intensify these systems and give rise to heavy antiviral weapons such as reactive oxygen species and nitric oxide. This results in the generation of an alliance against the viral enemy.

However, these heavy weapons have to be controlled to avoid too much harm to the host. By IL-4 and others, these reactions are hampered, but they are still allowed in foci of HSV replication, thus focusing the activity to only relevant sites. So, no hero does it alone. Rather, an alliance of cytokines, macrophages and other cells seems to play a central role. Implications of this for future treatment modalities are shortly considered.

## Introduction

Virus-host interactions are crucial for the outcome of infections. Several strategies have been utilized by viruses to overcome the host defence. For the virus to be successful, these evasive strategies have to be balanced with the pathology induced and the possibilities of transmission to new susceptible individuals. The mammalian host utilizes ubiquitous and redundant antiviral defence mechanisms. In different viral infections, different parts of the host defence seem to be crucial. However, the redundancy ensures that other systems are ready to take over, if one of them fails. The final outcome of a viral infection depends on a delicate regulation and timing of these antiviral effector mechanisms in response to the invading virus.

A viral infection of an individual thus involves a conflict between the virus and the host, which could conceptually be viewed upon as a human controversy escalating to invasion and armed struggle. To understand the resulting course of events it is important to know each party of the conflict and to conduct an analysis of the powerful weapons held by each of the combatants. The present review analyzes the early non-specific events in the conflict upon herpes simplex virus (HSV) infection. Initially, each participant of the conflict, the infecting HSV and the non-specific antiviral weapons of the host, are described. Subsequently, the early events of the conflict, the armament, early strikes and the opening battle between HSV and the host are discussed. Insight into the early non-specific defence mechanisms are important for our understanding of the conflict and may indicate how to intervene during serious systemic infections.

## The combatants – facts and hypotheses on function

### Herpes Simplex Virus

Herpesviruses are ubiquitous viruses generally infecting humans early in life. The majority of humans has had a primary infection with one or more herpesviruses and harbour these viruses in a latent state for the rest of their lives. The initial infection is most often asymptomatic, but can be symptomatic depending on the herpesvirus in question and the age and immune status of the host. The viruses are phylogenetically old and humans and herpesviruses have evolved together [1]. This co-evolution has created viruses which are well adapted to the human host and environment. Thus, herpesviruses are capable of coping with the human immune defence in a balanced manner generally without serious threads to the life of the host. Infection with a foreign herpesvirus, normally hosted by another species, does not always hold this balance, and the pathology is unpredictable. This is seen when humans are infected with the simian B virus, which often shows serious clinical outcome [2].

The human herpes simplex viruses were initially identified by Lowenstein, who passed it onto rabbits in 1919, and found it to be sensitive to alcohol and higher temperatures [3]. The viruses were classified into two serologically different types by Schneweiss in 1962 [4], and these are now known to belong to the subfamily of *Alphaherpesvirinae *together with varicella-zoster virus. These alphaherpesviruses all show neurotropic latency, and mucosal or skin lesions are frequently seen as a result of viral reactivation from sensory nerves. The two types of herpes simplex virus confer the genera *Simplexvirus 1 *and *-2*, which were formally designated by the International Committee on Taxonomy of Viruses as *Human herpesvirus *(HHV) *1 *and *2 *[5].

Herpes simplex virus (HSV) type 1 and type 2 are very closely related, showing a homology at the DNA level of 83% in protein coding regions and less in noncoding regions [6]. The genetic map of the two herpes simplex viruses is colinear [6], and the genomes are of approximately the same size, HSV-1 of 152 kbp [7] and HSV-2 of 155 kbp, and code for corresponding genes [6]. The minor sequence variations give different cleavage sites for restriction endonucleases, which has been used intensively as an important epidemiological tool [8-10].

#### Structure of herpes simplex virus

**Figure 1:**
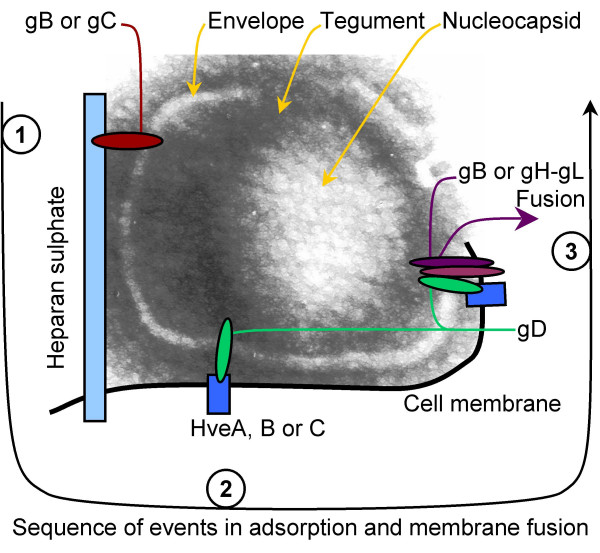
HSV composition and entry. Electron micrograph of negatively stained HSV particle with indications of major structural elements. Important mediators of adsorption to cells (1), receptor binding (2) and fusion of membranes (3) during the process of infection are drawn stylistically.

As all other herpesviruses the herpes simplex viruses are enveloped, icosahedral DNA viruses with a capsid of approximately 100 nm (fig. 1)[1]. The envelope holds at least 10 different glycoproteins protruding from the outer side (gB, gC, gD, gE, gG, gH, gI, gK, gL, and gM). The glycoproteins are primarily responsible for attachment to cellular receptors and fusion of membranes (especially gB and gD) [11-14]. In addition, there are two unglycosylated proteins in the viral envelope. The glycoproteins of the envelope have several immunoregulatory effects besides their primary more mechanical functions in viral attachment and entry [15-19].

In the space between the envelope and the capsid, the complete viral particles posses an almost amorphous structure which was termed the tegument by Roizman and Furlong [20]. The tegument consists of several viral proteins involved in the initial phases of viral infection and replication such as transport of the viral DNA out of the capsid [21], early shutoff of cellular protein synthesis (vhs) [22], and initiation of transcription of viral genes (α-trans-inducing factors) [23]. Besides the tegument seen in complete viral particles, tegument-like structures are seen in enveloped particles lacking a capsid and DNA, the so called light particles [24,25].

The capsid is composed of a complex icosahedral structure of 162 capsomeres, each with a central channel running from the outside to the interior of the capsid. Inside the capsid the double stranded linear DNA is packed as a spool with the ends in close proximity [21,26,27]. The genome consists of a long (L) and a short (S) segment which are covalently linked [28], and contains a high density of genetic information with about 94 open reading frames (ORF) and encodes approximately 84 polypeptides [7,29], of which only 37 are required for replication of the virus in cultured cells [30,31]. The viral genes are expressed in a cascade in groups classified as immediate early (IE, α), early (β), early late (γ_1_) and late (γ_2_) genes, each with a certain characteristic group of promoters regulating the sequential expression [29,32]. Generally, the α-gene products are transcription inducers, the β-gene products are viral enzymes such as the thymidine kinase and the viral DNA polymerase, and the products of γ-genes are the structural proteins of the viral particle [33]. The viral transcriptional chain is closed by some of the tegument proteins (e.g. VP16/Vmw65) which are γ-gene products with structural properties in the tegument of the viral particle and besides this harbour transcription-inducing capacity upon α-gene promoters crucial in the induction of the next replication cycle of the virus [32,34].

#### Infection of the cell

The HSV infection is initiated by adsorption of the viral particle via gB or gC to a cellular receptor, which is a heparan sulphate chain on cellular proteoglycans [35]. Thus HSV adsorption can be inhibited by heparin and soluble heparan sulfates [36,37]. This initial binding, in which gC is important but dispensable, is of greater significance for HSV-1 than for HSV-2, a divergence which could have implications for the different pathogenic patterns of the two strains [38,39]. Furthermore, trapping of HSV to heparan sulfate motives in the tissues, e.g. basal laminas, may be of importance for containment of the infection at a specific site [40]. Binding to the heparan sulfate-containing cellular receptors, which are in size with the HSV particle itself, is reversible, and serves to concentrate the viral particle in near proximity to the cell (fig. 1) [35,39,41].

A crucial step is then conducted by gD binding to an entry receptor, of which three classes has been described [42]. These include herpes virus entry mediator (HVEM), later designated as herpes virus entry protein A (HveA), which is a member of the tumour necrosis factor receptor family, nectin-1 (HveC) and nectin-2 (HveB), both members of the immunoglobulin superfamily, and heparan sulfate sites modified by 3-O-sulfotransferases [43-46]. The differential use of these receptors is of importance for HSV entry of different cell types and infection of polarized cells [47-51], exemplified by nectin-1, which is of importance in infection of the vaginal mucosa [52]. Upon binding to one of these entry receptors, conformational changes in gD lead to interaction with gB or gH-gL dimer, which results in membrane fusion by a mechanism not known in detail (fig. 1) [41,53]. The membrane fusion can take place both with the plasma membrane on the surface of the target cell and with an endosomal membrane after intraluminal pH-reduction, as it is seen for some other enveloped viruses [50,54-56].

Following these initial steps of infection several immunomodulatory cellular events are induced, but the potential importance of signalling through receptors involved in adsorption and membrane fusion is only scarcely analysed [57]. The receptor molecule HVEM is by its normal ligand capable of inducing activation of nuclear factor κB (NF-κB) and activation of T cells. By interaction with HSV-gD these receptor responses are inhibited. Thus, the HSV interaction with at least one of its receptors has multiple potentials for modulation of the host response to the infection [58,59].

#### Replication and formation of progeny virus

Upon fusion, the HSV nucleocapsid is transported by microtubules to a nuclear membrane pore where the viral DNA is released into the nucleus [60,61]. Both viral tegument products and cellular kinases are responsible for the initiation of α-gene transcription [62]. In these initial events the determination of whether it will lead to a lytic infection cycle or a latent infection seems to be directed largely by the infected cell type in question [63,64]. A key event in this seems to be early induction of latency-associated transcripts (LATs) with sequences antisense to the infected cell protein null (ICP0) and ICP4 [65-67]. In the initial phase of the lytic replication cycle, the IE-gene products, besides being transcription factors for the next wave of viral proteins, intimately regulate cellular functions in favour of viral replication and immune evasion [33,68]. Of these, the ICP0, a promiscuous transactivator without much DNA-binding capacity, forces the cell to a pre-dividing state optimal for viral protein synthesis [69,70]. Furthermore, ICP0 is active in inhibiting immune mechanisms such as interferon production and antiviral effects of interferons [71-73] and induces degradation of cellular proteins, involving the proteasome [74,75].

Very early in infection, the first transcriptional activity is seen just inside the nuclear membrane at the site were the viral DNA enters the nucleus [76]. The produced ICP0 co-localizes with the promyelocytic leukaemia (PML) nuclear bodies and initiates degradation of these, an event which seems to be important for productive replication of the virus [77,78]. ICP4 binds to parental viral DNA which is juxta-localized to the PML bodies, and later, when the bodies are degraded, replication compartments are formed, in which also ICP27 can be found [76,79,80]. ICP27 affects the posttranscriptional polyadenylation and splicing of RNA, and it is thus an element of the delayed host protein shutoff [81]. Immune evasion is additionally induced by the IE protein ICP47 which binds to transporter associated with antigen processing, TAP1/TAP2 and blocks the presentation of viral peptides by the major histocompatibility complex (MHC)-I [82].

The HSV progeny is formed in the nucleus of the infected cell, where the viral DNA is packed into preformed capsids. These are assembled with the tegument proteins and bud through the inner nuclear membrane to the perinuclear space [11]. The route of virus from here to the external side of the cell is controversial. Apparently two routes of viral egress are possible [11]. One way is by continuous passage through vesicles and the Golgi apparatus, where the membrane proteins are modified. The other route is by fusion of the newly acquired envelope with the outer nuclear membrane or the membrane of a vesicle, generating naked nucleocapsids in the cytoplasm. From here a new budding event should take place, for instance into the Golgi apparatus. The progeny virus thus acquires the envelope from other membranes, than the inner nuclear lamella, as it is indicated by analysis of membrane lipids [83]. Increasing evidence is pointing at this latter possibility of de-envelopment and re-envelopment as the dominating route of HSV egress [84-86].

The progress of HSV infection in tissues is influenced by the capacity of HSV to infect adjacent cells directly through cell junctions. The virus is thus avoiding exposure to extracellular substances such as antibodies and complement. The glycoproteins gE and gI are crucial for this kind of polarised transmission which primarily takes place in epithelial infections [47,87].

#### Epidemiology

As it is the case at the molecular level, the two herpes simplex viruses show similarities in their clinical appearance, both giving rise to primary infections of mucosal membranes and showing latency in sensory nerve ganglia [1]. The primary infections with HSV are often asymptomatic, especially at young age, but in a minority of cases vesicular or ulcerative lesions are seen. Although HSV-1 and -2 can give rise to indistinguishable clinical infections, there are differences in the anatomical distribution of these infections, as described in 1967 by Dowdle et al. [88]. HSV-1 is predominantly giving rise to infections above the waist, and HSV-2 to infections below the waist. This pattern is, however, not as straightforward as primarily described. In the last decades changes in both prevalence and distribution of HSV infections have been seen. The overall prevalence of HSV infection is very different in different countries and ethnic and social populations [89-91]. A decline in HSV prevalence has been observed in the western countries, probably because of improved socioeconomic conditions [92-94]. In parallel to the decline in prevalence, the aetiology of *herpes genitalis *has changed in several countries, presumably because of altered human habits and conditions of life [92]. In some areas of the world the proportion of genital infections caused by HSV-1 is still low (4–20 %) [95,96], but in others the relative proportion of genital herpes caused by HSV-1 is increasing [97,98]. In Norway, approximately half of primary genital HSV infections are caused by the type 1 virus [99], and in young women in Edinburgh, Scotland, 60% of new cases are caused by HSV-1 [100]. This shift of aetiology is probably caused by changes in sexual behaviour, especially oral-genital contact [101,102], and by the decreased prevalence of HSV-1 seropositivity at sexual debut, leaving a larger proportion of young adults permissive for a HSV type 1 infection [99]. Seropositivity to HSV-1 does not render any protection against catching an infection with HSV-2 [92,103,104], but a higher proportion of primary genital HSV-2 infections are asymptomatic in HSV-1 seropositive individuals than in seronegative individuals [103].

The aetiology of a genital infection is not insignificant, in that the frequency of recurrence is higher in HSV type 2-infected individuals than in those infected with type 1 [89,95]. The frequency of primary and recurrent infections with both HSV-1 and -2 has been reported to be higher among women than men [97,103,105]. Overall, these epidemiological changes could have implications for the risk of neonatal infection from vaginal delivery, in that more women are seronegative at delivery and thus a higher number have the risk of caching a primary HSV infection. On the other hand, less HSV is circulating, reducing the risk of those who are susceptible.

#### Clinical appearance and pathogenesis

As described above, primary infection with HSV is most often asymptomatic, especially in younger children [106]. However, some individuals experience a symptomatic primary infection with vesicular herpetic gingivostomatitis or in adolescence more often a pharyngitis [107]. As it is the case with orofacial infections, a primary genital HSV infection can be both asymptomatic and symptomatic with ulcerative lesions and with or without generalized symptoms such as fever, headache etc. [108,109]. Rarely, the infection disseminates to one or several organs giving rise to infections such as necrotising hepatitis, meningitis, encephalitis or to disseminated intravascular coagulopathy [110-113]. Such a clinical course, although uncommon, is most often seen in immunosuppressed patients e.g. transplant patients, neonates or pregnant women [114-116]. In pregnancy, primary infection with HSV without previous seroconversion at the time of delivery seems to be the main risk factor for infection of the newborn [109,117]. Genital HSV reactivations at labour only seem to posses a minor risk for neonatal infection of the baby [117,118], but in spite of this, approximately 70% of neonates infected are born by asymptomatic women [63]. The amount of virus in vaginal secretions during reactivations is much lower than the amount of virus in primary infections, and in reactivated cases maternal antibodies furthermore seems to be protective for the neonate [117,119-122].

When transmitted, the course of HSV infection in the newborn varies. In the pre-acyclovir era about one third of cases were mucocutaneus infections only involving the skin, mouth and eyes, one third were infections of the central nervous system (CNS) with or without mucocutaneus involvement, and the last third were disseminated infections involving multiple organs, including the liver, lungs, adrenals, and often the CNS [119]. Of these, neonates with a generalized infection had a one-year mortality of approximately 60%, those with CNS-infections had intermediary mortality, and nearly no mortality was seen in the group of patients with only mucocutaneus involvement [119]. In infected with multi-organ involvement the deaths are often set off by infection of liver or lungs or by coagulopathy. Sequelae, such as mental or neurological disabilities are seen in some of those with CNS involvement [123].

Now a day, after initiation of high-dose acyclovir treatment, the mortality and sequela rates have dropped [124]. The clinical pattern of neonatal HSV infections has changed in that less of the mucocutaneus infections disseminate to generalized infections when treated [123]. Even with high-dose acyclovir, improvements in treatment protocols are still needed, because the mortality is still as high as 30% in disseminated infections. Reduction in the time from debut of symptoms to initiation of therapy is vital and passive immunotherapy with HSV-specific antibodies could posses a potential as adjuvant to the antiviral treatment [123,125,126]. Other adjuvant treatment modalities are still needed in both neonatal infections and in generalized infections at later ages.

The pathology of HSV infections is mainly caused by a direct cytopathic effect of the virus, resulting in cellular lysis and focal necrosis of the infected area [119,127,128]. In tissues capable of regeneration, this is not devastating, provided that the lesions do not totally destroy the organ or result in functional disability during the infection. In the brain, however, the capacity for regeneration is small, and larger necroses induced by viral infection will result in life-long sequelae [119,123]. A delicate balance exists between the direct HSV-induced pathology and the immunopathology induced by immune reactions to the virus and the toxic and functional side effects of these reactions [129]. Immunopathogenesis seems to be the main aspect of HSV stromal keratitis, which often leads to blindness [130,131]. The scarification from this infection has even been attributed to autoimmunity by molecular mimicry [132]. Weak immune response to the virus leads to severe infections because of massive viral replication and dissemination. An immense immune reaction, especially with high amounts of virus to trigger a response, can bring about increased symptoms of infection, local symptoms such as high intra-cerebral pressure or pulmonary complications, as well as generalized or septic symptoms [129,133-136].

It is thus clear that early control of HSV replication in the initial phases of infection is crucial for the host. Early containment or at least inhibition of viral replication can prevent dissemination of the infection, and the early non-specific immune reactions thus have the potential to inhibit development of a symptomatic infection. Obviously the host will benefit from an attenuated or asymptomatic course of infection, but HSV – with the potential of subsequent reactivation from a latent site – could also benefit from such a course of infection, in that the host will survive and the activity of the host in society will not be hampered by symptoms from infection. Thus, the HSV has excellent chances to reach new susceptible hosts which bring the virus and the host in a situation of mutual benefit [33].

### Macrophages

Macrophages are ubiquitous cells of the mononuclear phagocyte system found throughout the body. Many attempts have been made to classify this range of cells with phagocytic activity. In 1892 Metchnikoff named them macrophages (large eaters) in contrast to microphages (the polymorphonuclear leukocytes)[137], and in 1924 Aschoff defined the reticuloendothelial system by the criteria of uptake of vital dye [138]. The macrophages are now more precisely defined as an important member of the mononuclear phagocyte system, defined in 1969 by van Furth and colleagues [139]. In the tissues they constitute a dynamic pool of cells with many functional capabilities, among which the capacity of phagocytosis, microbial killing, motility, and adherence to surfaces are classic [139].

The macrophages originate from the bone marrow, where proliferating promonocytes give rise to monocytes which enter the blood stream [140]. After a mean circulation time of approximately 11/2 day, the blood monocytes migrate to the tissues [140]. In the tissues the monocytes differentiate into macrophages with characteristics determined by the environment of the tissue in question [141]. The tissue macrophages in the major organs are represented by Kupffer cells in the liver, alveolar and interstitial lung macrophages, spleenic and sinusoidal lymph node macrophages, microglia in the brain, osteoclasts in bone, and Langerhans cells of the skin. Thus, macrophages are strategically situated all over the body taking care of debris from the organism itself and foreign material, among others invading microorganisms, including viruses [142,143]. Macrophages in different organs have different characteristics and functional capabilities and can not totally substitute one another in studies on macrophages [141,144-147]. Likewise, macrophages from different species can possess differences in their functional capability, e.g. the capacity for nitric oxide (NO) production [148,149].

Macrophages in tissues are, as described above, in part originating directly from monocytes, but they are also in part originating from local proliferation. This local proliferation in the tissues is performed by newly recruited monocytes, and in the steady state situation they only constitute a small fraction of the mononuclear phagocytes present [150]. Of the monocytes produced in the bone marrow of mice and passing through the blood, approximately half are targeting the liver, 15 % are going to the lungs, 25 % to the spleen and 7 % to the peritoneal cavity [150-152]. In the lungs, 70% of tissue macrophages in the steady-state originate from monocyte influx and 30% from local proliferation [153]. This proportion might vary between different tissues, as the lifespan of tissue macrophages in different organs also varies from around 6 days in mouse spleen to approximately one month for alveolar macrophages [151,152]. In the skin, Langerhans cells are a very stable and long-lived population of cells staying there for at least 18 month in the steady-state situation. However, in inflammation the Langerhans cells are within 2 weeks replaced and supplemented by circulating mononuclear cells [154]. When an inflammatory process is initiated, the dynamics of monocytes and macrophages are changed. Monocytes and other white blood cells are produced and recruited from the bone marrow, and the white blood cell count in the circulation is increased. The monocytes are mainly passing through the blood to become tissue macrophages, and the number of macrophages in the inflamed tissue can be increased by more than ten times [155]. In inflamed tissue the local proliferation of macrophages does not seem to increase, although the number of newly recruited cells is high, indicating that the differentiation of monocytes in the tissues is accelerated [155].

The differentiation of monocytes and activation of macrophages have been a focus of interest for many years because of the observation that macrophage activation is crucial in the defence against many intracellular pathogens [156-159]. It became clear relatively early that lymphocytes and soluble factors secreted by these (lymphokines) are important in activation of macrophages for killing of intracellular bacteria, e.g. *Listeria *[160]. In the killing of bacteria, interferon (IFN)-γ was shown to be an important stimulator of macrophage activation [161]. As mechanisms in performance of the killing simple toxic substances of reactive oxygen species (ROS) and nitric oxide were identified and seem to conduct their action in synergy [162-164]. The toxic substances are chemically simple, but their production and regulation in macrophages are very complex and still a matter of intense studies [149].

The state of the activated macrophage has changed conceptually from being viewed as one specific condition of the cell towards a more dynamic picture, provoked by the fact that macrophages activated by different means show different phenotypical characteristics [163,165]. The activated macrophage is now viewed as a cell with floating characteristics of many functional capacities regulated by a multitude of stimulating substances, such as the cytokine environment, hormones, and pathogenic and foreign substances [147,166]. Among variables, controlling macrophage activity in infected individuals, are the genetic constitutions of the host. The genetic background has been shown to be of importance for the regulation of both basic proliferation and function of macrophages and for the more specific antimicrobial responses [167,168].

### Cytokines

Soluble mediators of lymphocyte activities were described as early as 1953, but the first lymphokines/cytokines found and characterized were the type I interferons. Soon after, many other soluble mediators of lymphocyte and monocyte/macrophage activities were found [169-171]. The term lymphokine was introduced by Dumonde *et al. *in 1969, to describe lymphocyte derived factors, and the term monokine was used as a description of factors coming from the mononuclear phagocyte system, both acting on many cells, primarily leukocytes [172]. Because of a broader view on origin and function of these factors, the term cytokine is now more often used. Each cytokine was originally named according to biological activity in a functional assay, which often gave several different names to one cytokine, and thus confusion at the molecular level. To straighten this out, a numerical nomenclature of interleukins (between leukocytes) was introduced in 1979 [173]. This numbering system has clarified the field, but since it has no mnemonic functional anchorage it has drawn critique since then [174-176].

The cytokines are generally smaller proteins, some composed of two subunits, utilizing specific receptors on target cells for induction of their functional effects. They are structurally related in three families, with the prototypes being IL-1, IL-2 and IL-17 [176]. Functionally, cytokines are highly potent regulatory proteins acting in a paracrine or autocrine manner at picomolar concentrations [177]. The cytokine receptors are also structurally clustered in families, and functionally utilize a battery of overlapping kinases and nuclear binding proteins in their signalling pathway and thus have overlapping functions [178]. The final functional capacity of the effector cell thus reflects the cytokine environment experienced by the cell [177]. Thus the cytokines comprise a network of factors inducing or inhibiting each others secretion and function in different cells, giving rise to a constantly floating landscape of a large array of functional capacities [177]. In the early hours of a viral infection, the cytokines produced by cells infected or coming into contact with viral products are vital in conduction of the innate immune response to the infection [168,179].

#### Interferons

The interferons (IFNs) were described and named in 1957 by Isaacs and Lindenmann [170], who characterized the substances involved in the previously described interference of one virus with the replication of another unrelated virus, and the interfering activity of inactivated influenza virus with the subsequent infection of chorio-allantoic membranes [180-182]. The IFNs were the first cytokines described in detail, and thus provided the fundamental basis for the understanding of the cytokine concept [183]. The IFNs are divided into three major groups. The two original groups of IFNs are designated type I and type II, type I being the so called non-immune IFN, and type II the immune IFN. Type II (IFN-γ) is produced in high amounts as part of a specific immune reaction, whereas the type I IFNs can be produced by many cell types in response to, in immunological terms, non-specific stimulation. The many functions of IFNs and the growing understanding of signalling and regulation indicate that IFN analogues may play a major role in the next generation of new antiviral compounds [171].

The type I IFNs are a diverse group of cytokines, consisting of IFN-α, IFN-β, IFN-ε, IFN-κ, IFN-ω, IFN-δ, IFN-τ, and IFN-ξ/limitin [171,184]. The first five of these are expressed in humans, and their relative production depends on the stimulus and the cell type in question. The IFN-α family consists of multiple species and some of these in different allelic forms in both humans and mice. In humans 13 IFN-α genes and one pseudogene and in mice 14 IFN-α genes and 3 pseudogenes have been identified, clustering on chromosome 4 in mouse and chromosome 9 in man [185]. The functional importance of such a diversity is largely unknown. The subtypes differ in potency and have previously been shown to vary in their profile of activities [186,187], but new studies show correlation between antiproliferative and antiviral effects of various IFN-α species [185]. Thus, it seems that the importance of the diversity could come from varying expression patterns of the different IFN-α species. Most of the α IFNs are N-glycosylated, but glycosylation does not correlate with activity of the molecule, but rather with *in vivo *stability, and recombinant IFNs are shown to have activity comparable with that of the naturally produced molecules [185,188]. Only one IFN-β species exists, coded by a gene situated in the IFN type I cluster on chromosome 4 in mouse and chromosome 9 in man, as described above [185].

The natural IFN-α and -β have a molecular weight of 19 – 26 kDa and most species retain stability at pH 2 [189]. All type I IFNs bind to one common receptor composed of two subunits, IFN-α-receptor(R)1 and IFN-αR2. The IFN-α/β receptor (IFNAR) signal through the JAK/STAT-pathway by phosphorylation of the Janus kinase (JAK)1, tyrosine kinase (Tyk)2, signal transducer and activator of transcription (STAT)1 and STAT2, and induces genes with an IFN-stimulated response element (ISRE) in their promoter [171,190].

Generally the type I IFNs exhibit a huge range of biological effects, such as antiviral and antiproliferative effects, stimulation of immune cells such as T cells, natural killer (NK) cells, monocytes, macrophages, and dendritic cells, increased expression of MHC-I, activation of pro-apoptotic genes and inhibition of anti-apoptotic mechanisms, modulation of cellular differentiation, and inhibition of angiogenesis [171]. The newly discovered IFN-ξ/limitin also interacts with the IFN-α/β receptor, and is regarded as a type I IFN [184,191]. Antiviral activity of IFN-ξ has been shown against many viruses including HSV, and it exhibits both immunomodulatory and anti-tumour effects, but the lymphosuppressive activity is less than that of IFN-α [184,192]. A human homolog of IFN-ξ could thus have interesting potential in the therapy of tumours and viral infections.

The type II IFN is represented by only one member, the IFN-γ [193]. Structurally, IFN-γ is distinct from the type I IFNs, and it signals through a different receptor. For many years IFN-γ was thought only to be expressed by T cells. Later the large granular lymphocytes (NK cells) were recognised as important producers by the fact that Ia-antigen (MHC-II) expression on mouse macrophages could be induced by *Listeria monocytogenes *infection in SCID mice lacking T cells [194-196]. In recent years it has, however, been clear that other cell types, originally thought not to be producers of IFN-γ, are in fact capable of IFN-γ expression. So now macrophages, B cells, NKT cells and professional antigen-presenting cells are also recognized as IFN-γ producers in certain situations [197-202]. Induction and production of IFN-γ in antigen-presenting cells and NK cells seem to be vital in the early non-specific response to infections and of importance in the linkage to the adaptive specific responses coming up later [202-204]. The induction of IFN-γ production in non-T cells (e.g. NK cells) is conducted by cytokines, especially IL-12 in synergy with other proinflammatory cytokines, largely produced by mononuclear phagocytes [205,206].

IFN-γ exerts its effects through a distinct class II cytokine receptor, the IFN-γ receptor (IFNGR), composed of two subunits, IFN-γR1 and IFN-γR2. Upon binding of a homodimer of IFN-γ to the receptor complex, JAK2 autophosphorylates and then transphosphorylates JAK1. Activated JAK1 in turn phosphorylates IFN-γR1, which allows binding of the STAT1 homodimer to the receptor and subsequent phosphorylation of STAT1 [204]. The IFNGR and STAT1 are preformed as hetero- and homo-dimers, and upon receptor binding, the IFN-γ-IFN-γR1-STAT1 complex seems to be internalized and translocated to the nucleus, where the activated STAT1 homodimer binds to DNA at GAS elements and induces the first wave of responses [204,207-211]. Many of these initial IFN-γ induced products are transcription factors participating in further regulation of the many IFN-induced cellular response. Among these products are the IFN regulatory factors (IRFs) which stimulate or inhibit transcription of genes possessing an ISRE in the promoter region [204,212].

For many years the key mediator of macrophage activation during antigen-induced processes was recognised as macrophage activating factor (MAF) [213]. Only later, the crucial importance of these effects was attributed to IFN-γ [214,215]. IFN-γ has antiviral activity, but the most important effects of IFN-γ seem to be activation of macrophages, antigen-presenting cells, and NK cells and inhibition of T-helper type 2 (Th2) cells, resulting in a Th1-driven cell-mediated response to infection [204]. Experiments in knock out (KO) mice with deficient IFN-γ, IFNGR, or STAT1 expression have shown that this system is of major importance, but not vital, in the host response to viral infections [216-219].

Besides the two traditional groups of IFNs, a new group of IFN-like cytokines has been described in various species and named IL-28A (IFN-λ2), IL-28B (IFN-λ3), and IL-29 (IFN-λ1) [171,220]. These cytokines are antiviral proteins interacting with a distinct heterodimeric class II cytokine receptor composed of IFN-λR1 and IL-10R2, but sharing with the type I IFNs some intracellular signalling pathways through the ISRE [221]. Thus, they have a largely similar antiviral effect as the type I IFNs [220].

#### Tumour necrosis factor

Tumour necrosis factor (TNF, former designated TNF-α) and lymphotoxin (LT; former TNF-β) were for many years also known as cachectin from their involvement in cachexia of cancer patients [222]. TNF is a prototype and the second member of the TNF ligand superfamily (TNFSF2), now encompassing over 40 known signalling molecules, among which the LTα, LTβ, and LIGHT (*L*T-like, exhibits *i*nducible expression, and competes with HSV *g*lycoprotein D for *H*VEM, a receptor expressed by *T *lymphocytes) are some of the more prominent ligands [58,223]. Each member is the ligand of one or two distinct receptors of the TNF receptor family sharing a high degree of homology. The current nomenclature of these ligands and receptors has now been gathered on the internet [224]. TNF is a type II transmembrane glycoprotein coded from the human chromosome 6 and from chromosome 17 in mice [223]. It is synthesized as a 26 kDa transmembrane pro-TNF, primarily located in the membranes of the Golgi apparatus [225]. The pro-TNF is cleaved by a metalloprotease releasing the 17 kDa extracellular portion of the molecule [222,226]. Production and release of TNF from the cell is regulated at both the transcriptional and translational level and by post translational modification as described above [227]. During HSV infection both pre- and post-transcriptional regulatory mechanisms are involved in TNF production [228]. TNF is produced by many cell types of immune origin, primarily mononuclear phagocytes, neutrophils, NK cells and T cells, and has diverse effects on different cells [222].

Both membrane bound and soluble TNF interact as homotrimers with two different receptors, the p55 TNFR1 (TNFRSF1A) and the p75 TNFR2 (TNFRSF1B) [222]. As most other receptors of this family, TNFR1 holds a death domain important in the pro-apoptotic pathway. TNFR1 is expressed virtually on every cell type except erythrocytes, whereas TNFR2 is mostly expressed on endothelial and bone marrow derived cells [227]. The TNFR2 activates NF-κB (p50, p65/RelA, and p52/RelB) by ubiquitin-mediated degradation of inhibitor-κB (IκB) after phosphorylation by an IκB kinase (IKK). Besides inducing apoptosis, TNFR1 also activates NF-κB (p50/p65) [229,230]. Furthermore, the activator protein 1 (AP-1) is activated by mitogen-activated protein kinases (MAPKs) and together with NF-κB primarily acts in the proinflammatory pathways. Thus, signalling from the TNF receptor family induces a delicate balance between life and death (apoptosis) of the cell. Both of the TNF receptors can by proteolytic cleavage be converted to soluble receptors with the capacity to compete with their signalling ancestors, but also act to stabilize the trimeric TNF and thus maintain its activity [227,231].

The TNF superfamily seems to have evolved with the adaptive immune system in vertebrates and is crucial for the embryonic development of lymphoid tissue [223]. Furthermore, TNF is, as a proinflammatory cytokine, involved in activation of many immune cells and is thus an important factor of both the early non-specific and the specific immune response [232]. The importance of the TNF superfamily in antiviral defence is illustrated by the fact that different viruses have developed mechanisms for interference with nearly every step of activity of this system [227,229].

#### Interleukin-12, IL-23 and IL-27

IL-12 is the prime member of a small group of heterodimeric cytokines, all with the capacity to induce production of IFN-γ in a variety of cells. IL-12 was first described as an NK cell stimulatory factor (NKSF) and identified as a heterodimeric molecule composed of a p40 and a p35 subunit, which are covalently linked [233]. The p35 subunit has homologies to IL-6, and p40 is homologous to the extracellular domain of the haematopoietin receptor family, particularly the IL-6Rα chain [234]. The two IL-12 subunits are coded from different chromosomes, i.e. the human chromosomes 3 and 5 and the mouse chromosomes 6 and 11, respectively [235]. These genes are regulated separately, and coordinated induction in the same cell is required for secretion of the biologically active IL-12p70 heterodimer [236]. IL-12 is produced by monocytes, macrophages, dendritic cells, neutrophils and B cells [235,237]. In the initial response of spleen cells in mice injected *in vivo *with extracts of *toxoplasma gondii *or with lipopolysaccharide (LPS), the cellular source was found to be dendritic cells, but cultured macrophages have by themselves also been shown to produce IL-12p40 upon HSV-2 infection [238,239]. Such differences could depend on variations in the signalling mechanisms involved, which is also illustrated by the observation that the production in dendritic cells and macrophages has different kinetics. This difference could be brought about by differences in the requirement for co-stimulation with IFN-γ [240]. A collaborative action of dendritic cells and macrophages could be important, as indicated for IL-12 induction by influenza virus and other inducers [241].

The receptor for IL-12 is found on NK cells, T cells and dendritic cells and consists of two subunits (β1 and β2), which signal by the β2 subunit through the JAK/STAT pathway, primarily by activated STAT4 [235]. The primary effect of IL-12 is induction of IFN-γ production in NK cells and T cells, and IL-12 activates the cytotoxic potential of these cells. The IFN-γ locus in NK cells is constitutively demethylated and is thus ready for transcription of the gene, which is in contrast to that of T cells, [242]. Macrophages and NK cells are then stimulated by IFN-γ, resulting in activation for enhanced antimicrobial capacity [243,244]. IL-12 and IFN-γ in conjunction are the main responsible factors for activation of a Th1-driven adaptive cellular immune response, important for the long-term control of intracellular pathogens [235]. IL-12 stimulates proliferation of naïve T cells, and in conjunction with IFN-γ inhibits Th2 cell differentiation and the production of Th2 cytokines (e.g. IL-4, IL-5, and IL-13) [235]. Thus IL-12 holds a key position in induction and control of the Th1 response. The IL-12-induced IFN-γ production is synergistically enhanced by other cytokines such as TNF and IL-1 [240], and IFN-γ production can even be induced in macrophages by co-stimulation with IL-18 [197,245,246], a cytokine which by itself does not possess major IFN-γ-inducing capacity [240]. A positive feed-back loop is initiated by the IL-12-induced production of IFN-γ, in that IFN-γ is an important primer of IL-12 production, thus accelerating the system [247]. Furthermore, T cells enhance IL-12 production through signals of the proinflammatory TNF family [240]. In virus-infected macrophages a similar autocrine feed-back loop involving IL-12, IL-18, IFN-α/β, and IFN-γ could be speculated [248].

This potentially harmful situation, with accelerating IFN-γ production, regulated in a positive feed-back loop by IL-12, is inhibited by cytokines possessing anti-inflammatory properties. Among these IL-10 holds a crucial position as an inhibitor of IL-12 production, an effect which is also conducted by transforming growth factor-β (TGF-β) [249-251].The Th2 cytokines of the other side of the adaptive response, IL-4 and IL-13, inhibit IL-12 induction in the early phases of stimulation, but later they can be potent inducers of IL-12 production, although they still inhibit many of the IFN-γ-induced activities [212,252,253]. Phagocytosis of apoptotic cells by macrophages inhibits production of IL-12, a regulatory mechanism which seems to be important in restriction of the damages induced by uncontrolled defence mechanisms [254]. Injection of high doses of IL-12 to virus-infected mice is toxic, and leads to death with the pathology of TNF-related toxic shock, an effect which was explained by increased sensitivity to the toxic effects of TNF, and found to be dependent on the genetic constitution of the host [255,256].

The small IL-12 cytokine family also includes two other heterodimeric cytokines, IL-23 and IL-27, and a homodimer of IL-12p40. The latter is found *in vivo *in mice and functions as an antagonist of IL-12, but it is debated whether it exists in humans [257,258]. IL-23 is composed of the IL-12p40 and a p19 subunit and likewise binds to a receptor with one of the IL-12 receptor subunits (IL-12Rβ1) and a distinct IL-23R subunit [240,259]. The production and function of IL-23 is quite similar to that of IL-12, but IL-23 has a unique capacity to induce proliferation of memory T cells [235], and it has been found in nervous ganglia of HSV-infected mice on day 3 of infection [260]. IL-23 drives IL-17 production of NK cells, which mobilizes neutrophils and promotes production of the proinflammatory cytokines IL-1, IL-6, and TNF [261]. IL-27 is the newest recognized member of the family, constructed of two distinct subunits (EBI3 and p28), but still with functional capacities alike those of IL-12 [262]. The functional implications of these later discovered members of the IL-12 family is not yet clear, but it seems as if they are contributors to the overall effects of the IL-12 family and fine-tune the system [235,263-266]. The induction of IFN-γ and activation of NK cells is not only mastered by members of the IL-12 cytokine family. Other cytokines, like IL-15, are also implicated in development, function, and activation of these cells [267,268]. Generally, the IL-12 cytokine family has shown itself of importance in early defence against several viral infections, and as a vital inducer and regulator of the adaptive immune response against viruses and other intracellular pathogens [219,256,261,269].

#### Interleukin-4 and IL-13

Upon an accelerating pro-inflammatory response induced by initial viral replication the organism has to embank the IFN-γ-activated potentially harmful actions of macrophages and NK cells. Important mediators of this embankment are IL-4 and IL-13, which as described above repress the induction of IL-12, and thus put a brake on the positive feed-back loop of IFN-γ production [249,252]. Furthermore, IL-4 suppresses the production of other pro-inflammatory cytokines such as TNF and IL-1 [270]. Most importantly, IL-4 and IL-13 are potent inhibitors of the efferent arm of the pro-inflammatory system, and thus inhibit production of reactive oxygen species and nitric oxide. The production of these two potentially harmful effector mechanisms of activated macrophages is hampered by inhibition of production of the responsible enzymes in these reactions, the NADPH oxidase and the inducible nitric oxide synthase (iNOS) [271-273].

The primary producer cells of IL-4 and IL-13 are the Th2 cells, but these cytokines are also produced by basophils and mast cells [274-276]. The receptors for IL-4 and IL-13 are expressed on most cells and are composed as dimers of four different chains. IL-4 is the ligand of two receptors: A high-affinity heterodimer of IL-4Rα and the IL-2R common γ-chain and another heterodimeric receptor composed of IL-4Rα and IL-13Rα1. IL-13 binds to three complexes: A high-affinity heterodimer of IL-13Rα and IL-4Rα and two homodimers composed of either IL-13Rα1 or IL-13Rα2, which are both coded from genes on the human X-chromosome [276]. The immunomodulatory signalling is conducted through the JAK/STAT-pathway utilizing JAK1, JAK3 and STAT6. Phosphorylated and homodimerized STAT6 binds to STAT binding elements (SBE), which includes GAS, and either trans-activates or inhibits transcription of the adjacent genes [212]. The functions of IL-4 and IL-13 are nearly overlapping with only discreet discrepancies [276,277].

IL-4 was discovered in 1982 on the basis of another important effect of the cytokine, namely the ability to induce proliferation of B cells, and it was from this effect in the early years called B cell growth factor [278]. As this, some other effects of IL-4 are stimulating, in that it furthermore activates other Th2-like effects such as B cell class-switching and expression of mannose receptor and Fc receptor for IgE on macrophages [276]. Despite the anti-inflammatory profile IL-4 has *in vivo *been shown to confer some resistance to HSV infection [279,280]. IL-4 is thus not only an inhibiting cytokine but essentially an immunomodulatory cytokine with regulatory effects on macrophages as well.

## The armament and early strikes

The early innate defence mechanisms have for many years been regarded as important for the course of many viral infections, including infections with HSV [281]. The control of viral replication and dissemination during the first days of an HSV infection seems to be vital for the final outcome. If the viral replication is not halted by natural defence mechanisms during induction and maturation of the antigen-specific immune response, the adaptive immune system can be overwhelmed by massive viral infection at the dawn of activity of the specific reactions. The mechanisms of the anti-herpetic natural defence have been analysed extensively. It became relatively early clear that antiviral activity of macrophages [281] and NK cells [282] and early activity of the IFN-system [283] were important mediators of innate resistance to HSV. The relative contribution of each of these players in the early defence has been much debated, and as more interactions and molecular mechanisms are now elucidated, it seems clear that all of these players each hold a crucial position in an integrated antiviral natural defence system.

### Early induction of IFN-α/β by HSV

An important model used in the study of resistance mechanisms in defence against generalized infection with HSV is a mouse model, where mice infected intra-peritoneally or intra-venously experience a generalized infection with HSV replication in most organs, including the liver, spleen, and eventually the brain [284]. The dissemination of infection to the brain and the severity of infection of the peripheral organs depend in part on the age of the mice, as is the case in humans, where neonates have difficulties in controlling a HSV infection [281,285-287]. The course of infection in mice also depends on the type of HSV in question. Furthermore, in 1975 Lopez described a differential susceptibility of inbred mice to generalized infection with HSV, and this genetic difference in sensitivity has since been used for analysis of resistance factors of importance for the anti-herpetic defence [288]. In generalized infections, the genetics of the relative resistance to HSV-2 was shown to segregate with the X-chromosome [289]. This pattern of resistance to the generalized infection was for both HSV-1 and -2 attributed to a genetically determined difference in the capacity for IFN-α/β production [179,290,291], and it was shown that the X-linked pattern of resistance segregated with the HSV-2-induced production of IFN-α/β in macrophages during the first hours of infection [168]. Furthermore, macrophages from female mice respond to HSV with higher IFN-α/β production than macrophages from male mice [168]. This observation is in line with female mice being more resistant to HSV infection *in vivo *[291].

Early production of IFN-α/β has been correlated to resistance of HSV infections in several other studies. Treatment of mice with antibodies to IFN-α/β increases and accelerates mortality of a generalized HSV-1 infection and with higher doses of virus, mice are dying already after three to four days, a period where antigen-specific mechanisms are still in the induction and proliferation phase [292]. Furthermore, mice treated with mercuric chloride showed higher titres of HSV-2 in the first days of infection, an effect which could be correlated to impaired production of IFN-α/β [293,294]. In studies on peripheral HSV infections, such as cutaneous or corneal infections, IFN-α/β has been shown to be produced locally and to restrict the local replication of HSV and infection of nervous ganglia cells of the area, an effect which has also been correlated to the genetic constitution of the host [295-298].

The genetic background for the X-linked trait of HSV resistance and IFN-α/β production of macrophages remains unravelled. Induction of IFN-α/β upon HSV infection seems to be governed by different mechanisms in different cells [299]. IFN-α/β can be induced early by both infectious and UV-inactivated HSV in various cells, with the infectious virus being the more potent inducer in mouse peritoneal macrophages, whereas the UV-inactivated virus showed most potency in human peripheral blood mononuclear cells (PBMC) [168,299-302]. Production of IFN-α/β was induced by gD of HSV-1 in PBMC, but not in murine macrophages [17,303,304]. In PBMC-derived dendritic cells, however, the cellular mannose receptor was shown to be involved [299,305]. Furthermore, different Toll-like receptors (TLRs) have been shown to react with HSV [306]. TLRs are transmembrane pattern recognition receptors (PRRs) that detect redundant microbial molecular motives and induce antiviral and proinflammatory cytokines in response to alerting signals. In dendritic cells, TLR9-signalling, induced by the GC-rich HSV genome, has been shown to govern the induction of IFN-α/β, but TLR9-KO mice are still capable of controlling HSV infections *in vivo *[307-309]. However, in mouse macrophages the TLRs do not seem to be crucial for IFN-α/β induction upon HSV infection [304]. This is in agreement with the observation that the majority of IFN-α/β produced by spleen cells and dendritic cells and the total production from bone marrow macrophages was independent of TLR9 or MyD88, which is necessary for signalling by most TLRs [308]. In this study, heat inactivated virus was shown still to induce IFN-α/β in cells utilizing TLR9. As resident peritoneal macrophages do not produce IFN-α/β in response to even high doses of heat inactivated HSV, this gives an additional indication of independency from TLR9 of IFN-α/β production in macrophages [300]. Moreover, efficient induction of IFN-α/β by HSV in macrophages required dsRNA-activated protein kinase (PKR) activity and infectivity of the virus [304]. This is in agreement with the observation that dsRNA, which is produced by most viruses during replication, induces IFN through PKR, and not through TLR3, which also binds dsRNA [310]. Furthermore, another mechanism of IFN induction by dsRNA through a RNA helicase has been proposed [311].

The different induction patterns in different cells types, and the fact that IFN-α/β seems largely to be induced by other mechanisms than TLRs, explain the fact that knocking out TLR-signalling by MyD88 did not influence the *in vivo *infection with HSV in mice [309]. Other TLRs have also been shown to mediate signals in HSV infections. In HSV encephalitis in TLR2-KO mice, viral replication seemed unchanged or slightly increased during the first 4 days of infection, and the production of IL-6 and monocyte chemoattractant protein 1 were impaired, but interestingly pathological changes and mortality were reduced [134].

In relation to the X-linked resistance pattern of HSV infection and IFN production upon HSV infection, it is interesting that some of the TLRs are coded from the X-chromosome [312]. These are the TLR7 and TLR8, which are triggered by guanosine- or uridine-rich ssRNA in the endosomal compartment of cells [313,314]. There are, however, no indications that this pathway is implicated in IFN induction in cells during HSV infection, but the question has still not been directly addressed.

Regulation of the IFN-α/β gene induction is in part governed by activation of the transcription factors IRF-3 and -7, which are induced by IFN-α/β itself, resulting in a positive feed back loop, an effect which has been known for years without knowledge of the signalling mechanisms [315-317]. Thus, one possible explanation for the genetic differences in HSV-induced IFN-α/β production could be an elevated physiological level of this IFN self-stimulating system [318-321]. An analysis of the levels of IRF-3 and -7 in normal macrophages from these mice could be of interest. Analysis of the levels of the IFN-induced enzyme 2'-5'-oligoadenylate synthetase (OAS) in uninfected cells showed low but slightly higher levels in cells from relatively resistant mice [322]. With LPS, cells from the relatively resistant (C57Bl/6) mice show an early induction pattern of IFN-α/β, peaking within 2 hours, whereas cells from the susceptible BALB/c mice demonstrate a delayed response, peaking 7 hours after induction [323].

**Figure 2:**
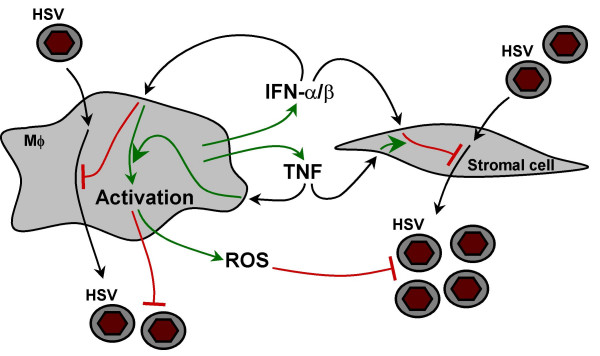
First early wave of response. The very early response to HSV infection of macrophages (Mφ). During the first few hours of infection HSV induces production of IFN-α/β and TNF in macrophages. The implications of these cytokines for HSV replication in neighbouring cells and for macrophage activation and production of reactive oxygen species (ROS) are outlined. Stimulatory pathways are indicated by green arrows (→), and inhibitory pathways are drawn in red.

Among other transcription factors involved in induction of the various IFN-α/β genes are the heterodimeric NF-κB family, which is activated by TLRs, IL-1R, and TNFR [324]. During a HSV infection NF-κB is activated and translocated to the nucleus [325]. Many regulatory mechanisms of NF-κB activation exist, one of them exerted through TNF, which is produced by macrophages very early during HSV infection (fig. 2) [293,300,325,326]. Thus, the responsible mechanisms might be exerted by other regulatory signals, influencing the magnitude of the HSV-triggered IFN-α/β induction pathway, and perhaps not by this pathway in itself [327].

A number of X-linked immunodeficiencies have been described, one of them being the Wiskott-Aldrich syndrome with defects in a protein expressed in haematopoietic cells, facilitating reorganization of the actin cytoskeleton, and thus influencing the mobility of immune cells and chemotaxis of macrophages. Patients with this X-linked immunodeficiency show aggravated herpetic infections, and cells from some patients seem to produce lower amounts of IFN in response to HSV [328-330]. Cells from patients with another X-linked immunodeficiency with mutations in the CD40-ligand, a member of the TNF family, showed decreased IFN-α/β production when infected with HSV-1, but these patients apparently show a normal response to viral infections [331,332]. This supports the notion above that other regulatory signals might be involved.

### Effect of early IFN-α/β on HSV replication

The overall effects of the IFN-α/β system, besides the production as described above, are determined by the sensitivity of cells to the secreted IFN-α/β. The effector mechanisms of IFN-α/β on HSV replication are not fully elucidated. Several IFN-α/β-activated systems are involved, including the dsRNA-activated PKR, which phosphorylates, and thereby inhibits, the elongation initiation factor (eIF)-2α, resulting in inhibition of translation [333]. Another important mediator of the antiviral activity is the OAS system, which activates 2'-5'oligoadenylate-dependent RNase L with the capacity to degrade single-stranded RNA [333]. Lately, the PML bodies have been described as crucial for the anti-HSV effect of IFN-α/β [334].

In mice exhibiting a relatively HSV-resistant phenotype, the direct antiviral effect of IFN-α/β in embryonic cells was found to be approximately three-fold higher than in cells from susceptible mice [322]. Data from another study showed comparable results on IFN-α/β sensitivity concerning the replication of encephalomyocarditis virus (EMCV) in cells from the same mouse strains [335]. This phenomenon was inherited as a co-dominant autosomal trait without any apparent influence of X-linked genes [322,336]. Further studies in mouse fibroblasts have revealed that TNF intensify the antiviral effect of IFN-α/β and, thus, the *in vivo *situation seems more complicated (fig. 2) [300,337]. In the original publication on genetics of HSV susceptibility in inbred mice, Lopez reported fibroblasts from the different mice to replicate HSV equally, and the same was found in the cells showing differential sensitivity to IFN-α/β [288,322]. In line with these results, the IFN-activated OAS, an inhibitor of HSV replication, was induced to a higher degree in cells from the resistant mice upon IFN-α/β treatment [322,333,338]. Furthermore, the level of stimulated and unstimulated OAS was generally found to vary between different inbred mouse strains [339]. Thus, the genetic difference in antiviral action of type I IFNs seems to affect the replication of several different viruses and to correlate with resistance to HSV.

The viral host protein synthesis shutoff, exerted by the HSV vhs-protein of the tegument, has major effects on the cytokine production of infected cells and reduces the effect of IFN-α/β on HSV replication [340]. Furthermore, the tegument proteins have been shown to induce cellular inhibitors of the JAK/STAT pathway, resulting in inhibition of both IFN signalling and production [341,342]. The IE protein ICP0 inhibits activation of IRF-3 and thereby also restricts IFN-induced pathways [71-73], and ICP0, ICP4 and ICP27 induce late shutoff of protein synthesis with decreased mRNA stability and thus reduced cytokine production [81,343]. As outlined, it thus seems HSV has evolved several mechanisms to evade the consequences of the IFN-α/β system, which underline the importance of these cytokines in the antiviral defence.

### Early effects of HSV on macrophage activation

During HSV infection macrophages are activated and possess an increased antiviral potential [281,344]. Classically, the macrophage antiviral activity has been described as intrinsic or extrinsic [345]. Resting macrophages possess a high degree of intrinsic activity against HSV, generally being non-permissive to viral replication. The macrophages are thus a blind end for the HSV infection, and they can in that way protect other cells from infection, for example as a barrier lining the liver sinusoids [344]. The extrinsic antiviral activity refers to the ability of macrophages to inactivate virus outside the macrophage itself or to inhibit viral replication in other cells [346]. The intrinsic antiviral activity depends among other factors on macrophage differentiation and has been correlated to IFN activity, either physiological levels of "spontaneous" pre-infection-synthesized or rapidly acting autocrine IFN-α/β [344]. In that respect, macrophages from mice of the resistant phenotype showed higher intrinsic activity by being less permissive to HSV replication [281,347].

One potential antiviral mechanism of macrophages may be the production of ROS. These were originally assigned to bacterial killing, but the effect of ROS has also been correlated to antiviral functions, although they might not be of major importance [348]. The ROS are mainly produced by NADPH-oxidases (Nox), which are membrane-bound multi-component enzymes primarily situated in the phagolysosome [349]. Activation of the NAHPD-oxidase, by phosphorylation and fusion of the enzyme subunits, primarily results in production of superoxide anion (O_2_^-^), which by superoxide dismutase can be converted to hydrogen peroxide (H_2_O_2_). The H_2_O_2 _in turn is then by Fe^2+ ^(Fenton reaction) or by Fe^3+ ^and O_2_^- ^(Haber-Weiss reaction) converted to hydroxyl radical (·OH), hydroxyl anion (OH^-^) and singlet oxygen (^1^O_2_), or by the myeloperoxidase to hypochlorous acid (HOCl) [349,350]. Small amounts of ROS are also produced by the mitochondria and may be of importance as signalling molecules from TNF [351,352].

During HSV infection *in vivo*, macrophages are activated and achieve an increased capacity to react with a respiratory burst of ROS when appropriately triggered, i.e. by phorbol esters (fig. 2) [353]. This macrophage activation is induced early in response to HSV infection, reaching a plateau within the first 12 hours of i.p. infection [353]. *In vitro*, macrophages were shown to be the cell type responding with an oxidative burst, and this capacity peaked after only 8 hours of infection with HSV [353]. This HSV-induced capacity for an increased respiratory burst was shown to be governed by autocrine IFN-α/β as a *sine qua non *phenomenon [300,353]. Nevertheless, TNF was also found to influence the macrophage activation. By itself, TNF reduced the macrophage capacity for a respiratory burst, but in combination with IFN-α/β it synergistically enhanced the IFN-induced activation [293,300]. Interestingly, a secreted portion of the HSV-gG acts as a phagocyte chemoattractant and induces production of ROS by signalling through the receptor activated by the phorbol esters [354].

The HSV-induced activation of macrophages *in vivo *is influenced by the genetic constitution of the host, with the most pronounced activation of macrophages originating from resistant mice, as expected on the basis of the genetics of IFN-α/β production in response to the infection. Furthermore, the genetics of the efferent part of the IFN-α/β-mediated HSV-induced activation of macrophages, displayed a co-dominant autosomal trait, as was the case with the antiviral effect of IFN-α/β in fibroblasts [336]. Thus, the genetically-determined sensitivity to IFN-α/β seems to be expressed in different cell-types. The influence of TNF on the genetics of this phenomenon has not been addressed. In Contrast to these observations, the genetics concerning the antiproliferative effect of IFN-α/β in bone marrow cells seems to be reversed [335,355]. This might, however, be linked, in that ROS are shown to activate various signalling molecules, mediate apoptosis, and exhibit antiproliferative effects depending on the dose and time of exposure [352].

Little is known on the potential antiviral effect of ROS. By examining peroxidized lipids, which is an oxidative product from ROS in tissues, it has been documented that these are produced during the acute HSV infection *in vivo*, and speculations on antiviral mechanisms have focused on induction of apoptosis [348,356,357]. HSV triggers apoptosis of infected cells by several pathways, and the importance of this phenomenon is indicated by the fact that the virus has evolved mechanisms to counteract each of these pathways [358]. Macrophages generally suppress apoptosis in HSV infections, as seen by increased apoptosis in macrophage-depleted mice [359].

Several studies on the mechanisms involved in the early battle against HSV, performed in *in vivo *animal models, have pointed to IFN-α/β as a crucial player. In adoptive transfer experiments, the effect of adult mouse spleen cells on the initial phase of a generalized HSV infection in suckling mice was conducted by IFN-α/β [360]. Furthermore, administration of a hematopoetic growth factor to neonatal mice increases the number of dendritic cells, B cells and NK cells, and confers resistance in a cutaneous model of HSV infection. The effect in this model could also largely be attributed to the actions of IFN-α/β, with some additional contributions by IFN-γ [361,362]. In KO-mice IFN-α/β was able to control the initial phase of a generalized HSV infection without contributions from NK, T- or B cells, but these latter players were necessary for survival and long term control of the infection [363].

The importance of an early, local IFN-response in models including *in vivo *progression and evaluation of final outcome of infection is more unclear, in that many other viral and host factors are of importance in these more complicated models with several stages of infection and involvement of different organs. Such models are, however, more close to the normal human HSV infection, starting at an epithelial surface, but to expect that one resistance factor in such a complicated system will come out clear as *the *responsible factor for the outcome downstream the sequence of events, is too simplistic. Nevertheless, induced expression of IFN-α/β in the eye by plasmid DNA or an adenovirus vector was shown to inhibit early local replication of HSV and the concomitant spread of virus to the brain and death from encephalitis [333,364], and in IFN-α/βR KO-mice HSV replicated to much higher titres than in normal mice [297].

This tells us that the innate and adaptive immune systems exhibit much redundancy, and that IFN-α/β is of vital importance in local inhibition of HSV replication. The multitude of antiviral mechanisms, be it innate or adaptive, have varying effects and importance in the different phases of infection, such as initial local infection, dissemination to other organs, establishment of latency and reactivation, and conclusions can not be drawn from one situation to another.

## The opening battle

The reactions discussed above, involving production of IFN-α/β and TNF, take place within the first 6 to 12 hours of a HSV infection, and thus are reactions, which can execute an effect within the first replication cycle of the virus. A little later, other cytokines such as IL-12, IL-18 and IFN-γ are produced and give rise to other weapons in the battle against the virus. They will, in turn, within the next replication cycle execute their actions, with potential harmful consequences for either parts of the conflict.

### IL-12 and IFN-γ production in early HSV infection

A few hours after the type I IFN and TNF response, macrophages react upon HSV infection with production of IL-12, which is seen from 8 to 12 hours after infection and on [238,365]. The same was found with other viruses 12 to 24 hours after infection [366]. In these and other studies, the producers of IL-12p40 during viral infection seem to be inflammatory cells, including macrophages, and not the infected stromal cells [365,367]. The IL-12 induction during HSV infection requires infectious virus, and it was shown to be regulated at the transcriptional level [238], as it is also the case when it is induced by LPS [247,367]. The dependence on infectivity is, however, in conflict with results from *in vivo *production of IL-12p40 and IFN-γ in draining lymph nodes from sites injected with UV-inactivated HSV [302]. High doses of UV-inactivated virus were used, and some minimal transcription of viral genes could have taken place, although the virus was not replication competent. Transcription of the IL-12p40 gene in macrophages requires *de novo *protein synthesis during the inducing HSV infection, which could explain the relatively late appearance of IL-12 production [238,367]. The κB-sequence of the IL-12p40 promoter binds NF-κB in HSV-infected cells, and the production of IL-12p40 was found to be repressed by an inhibitor of NF-κB activation [238]. Both these observations indicate that signalling through NF-κB is of significance in HSV-induced IL-12 production.

In human macrophages, TNF has been shown to inhibit IL-12p40 production, but not p35 production, by a mechanism not involving NF-κB [368]. Furthermore, IL-12 has in a mouse model been shown to stimulate TNF expression [255], indicating that TNF can participate in a negative feed-back loop in the regulation of the IL-12 system [369]. Likewise, IFN-α/β has been shown to inhibit IL-12 production in both humans and mice [370-372]. The implication of such inhibition by IFN-α/β and TNF, which are secreted very early in HSV infections, well before the production of IL-12, has so far not been elucidated.

As described earlier, the IL-12p40 induction is influenced by IFN-γ in a positive feed back loop. IFN-γ could activate IL-12 transcription through binding of IRF-1, -2, and -8 to an ISRE site in the promoter-region of IL-12 [373,374]. Upon HSV infection, IFN-γ is produced as part of the non-specific response to the virus. A marked synergism between HSV and IFN-γ in IL-12 induction has been demonstrated [238], indicating that the IL-12 / IFN-γ auto-accelerating system is of importance during HSV infections.

The IFN-γ-inducing activity of the produced IL-12 is pronounced in mouse peritoneal cells after 24 hours of infection with HSV [238]. In a study by Kirchner et al. IFN-γ was detected as early as on day 3 of *in vivo *HSV infection, and the IFN-γ production was correlated to the genetics of HSV resistance [375]. During HSV infection, the production of IFN-γ is mainly induced as a concerted action of several factors and not by IL-12 alone. IFN-α/β by itself was shown to be a weak inducer of IFN-γ production by NK cells, but in synergy with IL-12 the production of IFN-γ was markedly enhanced [376]. In elicited peritoneal macrophages, HSV induced efficient IFN-γ production through cooperation of IL-12, IFN-α/β and IL-18 [377]. In such a proinflammatory environment even other cells than NK and T cells, e.g. macrophages, might produce lower levels of IFN-γ [200,202,245]. IL-12 signals through STAT4, but STAT4 translocation to the nucleus of NK cells has also been seen after IFN-α/β stimulation [206,378]. Likewise, IFN-α/β induces STAT4 phosphorylation in T cells [379], indicating that IL-12 and IFN-α/β at this point act through a shared signalling pathway. Furthermore, the synergistic action of IL-12 and IL-18 in IFN-γ production by macrophages was shown to be dependent on STAT4 [197]. In addition to these factors, TNF and IL-1 have also been shown to act in synergy with IL-12 in IFN-γ induction [206,380,381] and *vice versa*, IFN-γ has been shown to synergize with HSV in induction of TNF production [325]. This further emphasizes the concept of positive feed-back mechanisms in the regulation of early IFN-γ production.

**Figure 3:**
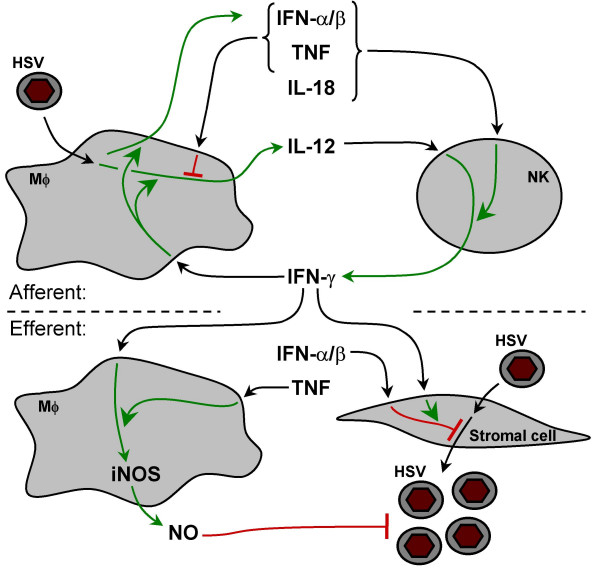
Second early wave of response. Regulatory pathways controlling production and action of IFN-γ during early HSV infection. When infected with HSV macrophages (Mφ) produce several cytokines, including IL-12, which stimulate production of IFN-γ, primarily in NK cells. IFN-γ then induces NO production in macrophages and stimulate the direct antiviral activity of IFN-α/β in other cells. Stimulatory pathways are indicated by green arrows (→), and inhibitory pathways are drawn in red.

The important direct effect of IFN-α/β on HSV replication was found to be enhanced synergistically by IFN-γ in both cell culture and *in vivo *in mice [363,382-384]. This is, however, in conflict with an early study, which could not reveal any synergism between IFN-α/β and IFN-γ on the replication of HSV in human blood mononuclear cells [385]. Synergistic action of the two types of IFN is further supported by the observation of synergism between IFN-γ and TNF on HSV replication in corneal cells, and the fact that this was exerted through production of IFN-β [386-388]. The effect was, however, greatly dependent on the cell type examined, which could explain the above-mentioned inconsistency. Synergism between TNF and IFN-γ in inhibition of HSV replication has now been shown to be mediated by activation of a tryptophan-depleting enzyme [389]. Thus, relatively small amounts of early IFN-γ produced by NK cells in response to IL-12, IFN-α/β, TNF, and IL-18 could in collaboration with the already present IFN-α/β and TNF have important local effect on HSV replication in permissive cells (fig. 3). This conclusion is further supported by observations in KO mice, indicating that collaborated action of IFN-α/β and IFN-γ is of importance in control of subcutaneous HSV infections [362].

*In vivo *studies on HSV infections in immunodeficient, KO, and antibody-treated mice have shown that the IL-12, -23 / IFN-γ system is able to control the infection, affecting both the survival rate and the HSV titres early in infection [390,391]. The effect of IL-12 in HSV infections seems to be conducted in synergy with IL-18 [390], as it has also been shown for vaccinia virus [392]. In HSV corneal infections in KO mice, IL-12 was shown to participate in the immune pathogenesis [393], but in another study utilizing IL-12 encoding plasmid DNA, corneal expression of IL-12 reduced the angiogenesis, and thus the pathology of the infection [394]. However, both studies agreed that IL-12 does not affect the local titres of HSV in the eye. After a thermal injury, wide-spread HSV infections are an important risk, and treatment of injured mice with IL-12 combined with soluble IL-4R results in augmentation of the IFN-γ production and decreased viral replication and mortality [395].

In mice infected with murine cytomegalovirus (MCMV) production of IL-12-induced IFN-γ by NK cells has been demonstrated *in vivo*, and the system was further shown to lower the viral titres [396,397]. The IL-12 / IFN-γ system seems, however, not to be of importance in all viral infections, in that the latter study could not detect any production of early IL-12 or IFN-γ in a model of infection with the arenavirus lymphocytic choriomeningitis virus. Analyses of the IL-12, -23 / IFN-γ system in humans with genetic defects and in KO-mice reveal more redundancy in man than in mouse and indicate that the system is of more importance in DNA- than in RNA-virus infections [219].

The producers of early IFN-γ, the NK and NKT cells, and the cytokine IL-15 and the transcription factor T-bet, which are both crucial for the differentiation and function of these cells, have all been shown to be decisive for the early control of HSV infection *in vivo *[268,398-400]. Although NK cells but not IFN-γ was shown to be decisive for survival from ocular infections [401], such an effect of IFN-γ has been seen by others [402]. Furthermore, a review of genetic functional NK cell defects found NK cells and their innate IFN-γ production to be of central importance in herpesvirus infections [403].

Overall, it can be concluded that the IL-12 / IFN-γ system is active in HSV infections and possesses an important antiviral potential, capable of controlling viral replication during the early phases of infection.

### Production of NO in early HSV infection

In macrophages exposed to IFN-γ, the enzyme inducible nitric oxide synthase is induced, which eventually results in production of NO from molecular oxygen and a guanidino nitrogen by conversion of L-arginine to L-citrulline [404]. Upon HSV infection, the iNOS gene is induced, as shown by detection of iNOS-mRNA in infected mouse peritoneal cells and corneal neutrophils [405,406]. The production of NO in HSV-infected cultures of resting mouse peritoneal cells, which comprise a mixed population of macrophages, lymphocytes, NK cells etc., is dependent on the virus being infectious [405]. This is in line with the requirement of infectious HSV for IL-12 production and thus for production of IFN-γ as described previously [238]. NO could itself be involved in a positive feed-back, in that signalling of IL-12 utilizing Tyk2 requires the activity of NO [407]. When exogenous IFN-γ is added to virus-infected cells, a marked synergism is seen. This synergistic effect of HSV on the IFN-γ-induced NO production in macrophages was shown to be mediated by autocrine secretion of TNF [325,405]. In line with this, mice with a targeted disruption of the TNF gene showed impaired resistance to HSV and increased viral replication within the first days of infection [408], and antibodies to TNF and an inhibitor of NO production impaired early control of HSV infection in peripheral nervous tissue [409].

The induction of iNOS and the following production of NO in response to IFN-γ and HSV is a relatively slow reaction, coming up after about 18 hours of infection [405]. In *in vivo *vaginal HSV infections iNOS mRNA could be detected after 24 hours of infection [410]. Thus, the production of this relatively toxic substance is part of the second wave of innate defence mechanisms. The retarded production of NO and the requirement for two or more signals for induction of iNOS are logic considering the toxicity of NO and the potentially harmful consequences for the host.

In HSV-infected macrophages exposed to IFN-γ, iNOS is induced synergistically though TNF-induced NF-κB activation and translocation to the nucleus, as shown by binding of a heterodimeric complex of p55/p65 and a homodimer of p55 to the κB-site of the iNOS promoter during infection [325]. The crucial position of NF-κB in the induction of iNOS and production of NO is also indicated by experiments showing that antibodies to TNF inhibit activation of NF-κB and production of NO in HSV-infected cells and abolish the synergism between the virus and IFN-γ, an observation which was also seen with inhibitors of NF-κB activation [325]. Further analysis of the signalling mechanism has revealed that the synergism upon HSV infection is influenced by physical interaction of IRF-1 and the NF-κB subunit p65 and controlled by the ISRE-site and the distal κB-site of the iNOS promoter (fig. 5) [411]. A further support for this notion comes from the observation that the DNA-binding capacity of NF-κB and the nuclear translocation of IRF-1 have similar kinetics upon HSV infection [411] and the fact that IRF-1 is essential for iNOS induction [412]. Induction of other genes such as IFN-β and vascular cell adhesion molecule 1 also involve physical interaction of IRF-1 and NF-κB [413], and both IRF-1 and IRF-2 have in other cells types been shown to form complexes with NF-κB [414,415]. Another potential mechanism in the synergistic induction of iNOS could involve complex formation of IFN consensus sequence-binding protein (ICSBP or IRF-8) and IRF-1, which is also important for high-output NO production but has still not been studied in HSV infections [416].

**Figure 4:**
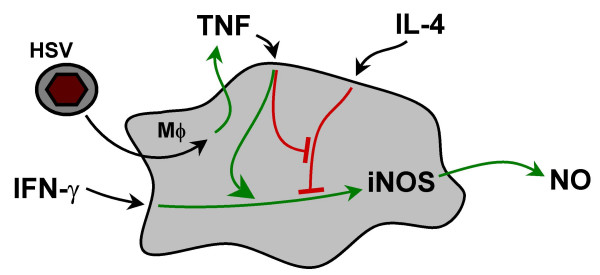
Regulation of iNOS induction at the cellular level. Cytokines controlling the iNOS induction in macrophages (Mφ) during early HSV infection. IFN-γ, produced mainly by NK cells, stimulates iNOS production. This IFN-γ-induced production of iNOS can be inhibited by IL-4. Upon HSV infection of macrophages they produce TNF which synergizes with the IFN-γ-induced pathways and inhibits the inhibitory signals of IL-4. Thus, the virus overrules the restrictive signals and opens up for an otherwise closed pathway. Stimulatory pathways are indicated by green arrows (→), and inhibitory pathways are drawn in red.

**Figure 5:**
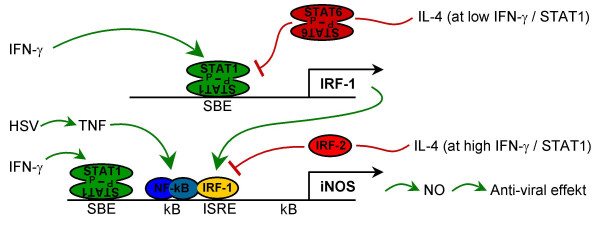
Regulation of iNOS induction at the molecular level. Transcription factors controlling induction of the iNOS gene. Activated STAT1 induces transcription of the IRF-1 and iNOS genes, an effect which is competed by activated STAT6. IRF-1 interacts physically with NF-κB, binds to the distal κB-binding site of the iNOS promoter region, and stimulates transcription. Only when NF-κB is absent, IRF-2 can bind to the ISRE site and block transcription. Stimulatory pathways are indicated by green arrows (→), and inhibitory pathways are drawn in red.

Thus, high-output NO production from activated macrophages is controlled by a "double-lock" signalling mechanism restricting the production of this antiviral toxic substance to sites of active viral replication, and sparing uninfected tissue from the detrimental effects (fig. 4).

The antiviral effects of NO have been documented in several viral infections, although there clearly exist viruses and conditions where NO does not exhibit major antiviral properties. NO is thus not a magic bullet against virus infections [417]. In HSV infections, NO has been shown to confer a substantial part of the antiviral activity induced by IFN-γ in a macrophage cell line and to participate in the extrinsic anti-HSV effect of macrophages [418-421]. An exogenously added donor of NO has in several cell lines been shown to reduce the replication of HSV [422]. *In vivo*, analysis of mice treated with an inhibitor of NO production showed higher titres of HSV in the lungs but increased survival rates due to reduced inflammation [135]. Recently, a study using another inhibitor of NO production has confirmed the anti-herpetic effect of NO during a HSV respiratory infection, but in this study mice with inhibited NO production showed increased inflammatory responses, symptoms of infection, and mortality [423]. Replication of HSV during vaginal infection was increased in the presence of an inhibitor of NO production, and this enhanced viral replication was most prominent during the first 24 hours of infection [410]. In iNOS-KO mice, the herpes virus MCMV replicates to higher titres in various organs and in macrophages, and this results in impaired survival of the animals [424]. Weanling mice with a targeted disruption of the iNOS gene showed increased HSV replication, but apparently without differences in HSV titres during the first days of infection [425], and in adult KO mice, we could not detect any significant effect of NO during the early days of a generalised HSV infection (Ellermann-Eriksen, unpublished results). Probably, these *in vivo *results are due to redundancy of the antiviral system. [426].

The final effects of NO on HSV infections therefore appear to be balanced between antiviral versus toxic effects, and the final outcome seems to depend on the timing, infectious dose, and tissues involved. Thus NO production in the early phases of HSV infection is one of the effector mechanisms of the innate immune response inhibiting HSV replication, but when overproduced, NO might itself result in pathology, as discussed in the following section.

### Restriction of NO production during HSV infection

As outlined above, positive feed-back mechanisms exist at the afferent side of the early cytokine response, involving especially the production of IFN-γ, IL-12, IFN-α/β and TNF, and synergisms at the efferent side, resulting in high-output NO production. As a result of coordinated induction of the iNOS gene by several transcription factors, activated by especially IFN-γ and TNF, a potent early antiviral system is activated. However, NO causes damage to DNA, proteins and lipids in cells and tissues and could thus be deleterious for the host [427-430]. A study in KO-mice indicates that NO can be responsible for inflammation and life-threatening symptoms to HSV infection of the lungs [135]. This effect of NO on pulmonary symptoms is also observed in influenza virus infections [431], although NO inhibits replication of both influenza virus and severe acute respiratory syndrome coronavirus [432,433]. Consequently, when this system is activated, it has to be controlled and eventually closed down, as it would otherwise induce unnecessary harm to the host. Such negative regulations of the iNOS gene induction in IFN-γ activated macrophages is conducted by IL-4 and IL-13 [272,273,434]. Furthermore, TGF-β can exhibit down-regulation of NO production through several post-transcriptional regulatory mechanisms, but the contribution of these pathways have not been analysed in HSV infections [435,436]. IL-4 production during HSV infection has in vaginal and CNS infections been demonstrated on day 2 of infection and to increase for the next days [437,438]. In peritoneal cells from mice infected i.p. production of IL-4 could be detected at day 5 of infection [439].

At low IFN-γ concentrations, IL-4 has been shown to inhibit iNOS induction through STAT6 competition with STAT1 binding to the GAS element of the IRF-1 promoter region. This results in reduced expression of the transcription factor IRF-1, which is crucial for induction of iNOS [440]. Generally, STAT6 was shown to be a key factor in IL-4- and IL-13-induced inhibition of iNOS gene transcription induced by IFN-γ (fig. 5) [441].

At higher IFN-γ concentrations, activated STAT6 is no longer able to compete with the high amounts of activated STAT1 dimer [440]. However, in this situation IL-4 is still able to inhibit the production of NO from IFN-γ-stimulated macrophages [272,273,434]. In the presence of high levels of IFN-γ, IL-4 is not able to alter the induction of IRF-1, but the production of IRF-2 is increased [434]. The human promoter region of IRF-2 contains a SBE, and the induction of IRF-2 could thus potentially be mediated by STAT6 binding to this element [442]. This is in agreement with the fact that IRF-2 is known to compete with the binding of IRF-1 to ISRE sites and to antagonize the trans-activating activity of IRF-1 in the regulation of other IFN-induced genes [443-445]. Inhibition of iNOS expression by high concentrations of IRF-2 relative to IRF-1 has thus been proposed as a controlling mechanism in situations with high levels of IFN-γ (fig. 5) [434]. Furthermore, another mechanism could evolve from the observation that IL-4 signalling can result in disruption of the complex formation of ICSBP and IRF-1 and thereby inhibit iNOS induction [416]. Other mediators of IL-4-induced repression of iNOS induction might exist, in that another DNA-binding transcriptional repressor competing with IRF-1 has been described [446].

In IFN-γ activated macrophages the IL-4- and IL-13-induced inhibition of iNOS induction can thus be overruled by HSV infection, leading to a sustained NO production (fig. 4) [439,447]. This effect of HSV infection is mediated through TNF production and NF-κB activation [439,447]. However, pre-treatment with IL-4 has in a Theiler's murine encephalomyelitis virus model showed inhibition of NF-κB activation [448]. In thioglycollate-induced peritoneal cells, LPS and TNF could only overcome the inhibiting effect of IL-4 in situations, where IL-4 was added simultaneously or after the stimulators [272], a sequence of events which, however, is in agreement with the sequence of cytokine production in HSV infections. When activated, the NF-κB p65 physically interacts with IRF-1 and trans-activate iNOS transcription in HSV-infected and TNF-treated cells [411,449]. It is thus tempting to speculate that the NF-κB-IRF-1 complex has higher affinity for the combined DNA-binding site and thus is able to obstruct the binding of IRF-2 to the ISRE site of the iNOS promoter and in that way turn the competition towards transcriptional activity (fig. 5) [411]. This will block the inhibiting effect of IL-4 in foci of HSV replication and open up for NO production at sites where the antiviral effect is of more importance than the potential toxicity.

## Conclusions and perspectives for future clinical intervention

In treatment of HSV infections, we have for many years had a very powerful tool in the antiherpetic drug acyclovir and related compounds. But there are still therapeutic problems in the group of patients with generalized or CNS infections, and therefore it is tempting and timely to hypothesize on possible future treatment strategies. As described, it is clear that relatively discrete but early actions of the non-specific defence systems are crucial for the long term outcome of the infection. The same holds for the antiviral therapy, and early presumptive therapy and rapid diagnostics could thus potentially improve the final outcome. In the seeking for improved antiviral treatment, adjuvant therapy with anti-HSV antibodies could potentially accelerate the clearance of viral particles, and block viremic dissemination in patients, who are still seronegative at the time of treatment.

Immunomodulatory treatment modalities imitating the early non-specific antiviral defence, working as described in this review, could be considered. The key players exhibiting the least toxicity by themselves could be used, taking advantage of potential synergy with other cytokines in the foci of HSV infection. In the future, molecules with affinity for various receptors are expected to be produced, and when we know the signalling mechanisms in detail and all the potential interactions, molecular signalling could be addressed directly by pharmaceuticals.

In consequence of the crucial position of the type I IFNs in innate response to HSV, future analogues of IFN-α/β seem obvious as candidates for adjuvant treatment of severe HSV infections. This could be supplemented with IL-12, which would give the highest IFN-γ production in foci of HSV infection because of other cytokines such as IFN-α/β, TNF and IL-18 being present there. With focussed production of IFN-γ at sites of active viral replication and treatment with IFN type I analogues the focal antiviral activity could be increased markedly, without too much activity in areas without infection. To hamper systemic consequences of the enhanced proinflammatory reactions, such pro-inflammatory treatment could perhaps benefit from concomitant treatment with IL-4 or other STAT6-activating therapeutics in the future. This would further focus the activity to sites of active HSV replication. In situations with massive viral replication in nearly all organs, high-dose aciclovir should perhaps only be supplemented with anti-inflammatory medications and inhibitors of TNF, since many of these individuals risk to die from septic reactions.

## Competing interests

The author(s) declare that they have no competing interests.
